# Increased Prediction Accuracy Using Combined Genomic Information and Physiological Traits in A Soft Wheat Panel Evaluated in Multi-Environments

**DOI:** 10.1038/s41598-020-63919-3

**Published:** 2020-04-27

**Authors:** Jia Guo, Sumit Pradhan, Dipendra Shahi, Jahangir Khan, Jordan Mcbreen, Guihua Bai, J. Paul Murphy, Md Ali Babar

**Affiliations:** 1 Department of Agronomy, University of Florida, Gainesville, FL USA; 2 USDA-ARS Central Small Grain Genotyping Lab, Manhattan, Kansas USA; 3 Crop and Soil Sciences, North Carolina State University, Raleigh, North Carolina USA

**Keywords:** Genetics, Plant sciences

## Abstract

An integration of field-based phenotypic and genomic data can potentially increase the genetic gain in wheat breeding for complex traits such as grain and biomass yield. To validate this hypothesis in empirical field experiments, we compared the prediction accuracy between multi-kernel physiological and genomic best linear unbiased prediction (BLUP) model to a single-kernel physiological or genomic BLUP model for grain yield (GY) using a soft wheat population that was evaluated in four environments. The physiological data including canopy temperature (CT), SPAD chlorophyll content (SPAD), membrane thermostability (MT), rate of senescence (RS), stay green trait (SGT), and NDVI values were collected at four environments (2016, 2017, and 2018 at Citra, FL; 2017 at Quincy, FL). Using a genotyping-by-sequencing (GBS) approach, a total of 19,353 SNPs were generated and used to estimate prediction model accuracy. Prediction accuracies of grain yield evaluated in four environments improved when physiological traits and/or interaction effects (genotype × environment or physiology × environment) were included in the model compared to models with only genomic data. The proposed multi-kernel models that combined physiological and genomic data showed 35 to 169% increase in prediction accuracy compared to models with only genomic data included when heading date was used as a covariate. In general, higher response to selection was captured by the model combing effects of physiological and genotype × environment interaction compared to other models. The results of this study support the integration of field-based physiological data into GY prediction to improve genetic gain from selection in soft wheat under a multi-environment context.

## Introduction

Genomic selection (GS) that predicts genomic estimated breeding value (GEBV) of individuals using genome-wide molecular markers^1^ has proven to be a promising technique for accelerated plant breeding. Studies have shown that breeding programs that incorporated GS often resulted in a near two-fold genetic gain compared to standard phenotypic selection^2,3^. The rapid development of high-throughput phenotyping in multi-environment field trials and multi-variate statistical tools also contributed to improved accuracy of prediction and selection of candidate lines^4,5^.

In GS, a training population (TP) is established to estimate marker effects, for which phenotypic (e.g. grain yield) and genotypic (e.g. DNA marker) data are available. The estimated marker effects from the TP are then used to predict phenotypes in a new set of germplasm, called breeding population (BP) or validation population (VP), that only need to be genotyped with DNA markers. A breeding value will be predicted for all the individuals in BP based on the composition of markers being scored. Individuals with high GEBV will be selected prior to being evaluated in field experiments, therefore, increasing selection population size and accelerating the selection-evaluation cycles in plant breeding^6,7^. GS is particularly valuable for many quantitative traits such as grain yield and biomass partitioning traits that are affected by large numbers of small-effect genes^8^. Therefore, the selection of the complex traits using genome-wide markers can be more effective than marker-assisted selection using a few markers.

The importance of exploiting multi-environment information has been recognized in plant breeding to overcome genotype × environment interaction (GE). Burgueño *et al*.^9^ evaluated three crops (potato [*Solanum tuberosum* L.], maize [*Zea mays* L.], and wheat [*Triticum aestivum* L.]) in a multi-environment trial and concluded that the predictability of the model increased up to 6% when GE was included in a factor analytic model. Another study in maize (*Zea mays* L.) showed similar results when GE effect was modeled to account for the heterogeneity and correlation between environments^10^. In recent years, combining phenotypic and genomic data in prediction studies have emerged as a useful technology for improving breeding efficiency. Montesinos-López *et al*.^11^ used hundreds of reflectance data from hyperspectral cameras to predict wheat grain yield. They found that using all reflectance data simultaneously increased prediction accuracy than using a single vegetation index alone. In another study, Aguate *et al*.^12^ indicated that integrating all hyperspectral wavelengths using ordinary least squares, partial least squares, and Bayesian shrinkage resulted in higher prediction accuracy than using individual vegetation indices in maize. Pérez-Rodríguez *et al*.^13^, Cuevas *et al*.^14^, and Crain *et al*.^15^ reported improvement in prediction accuracies using a multi-environment model relative to a single-environment model. Montesinos-López *et al*.^16^ also observed that prediction models incorporated hyperspectral data and spectrum by environment interaction terms were more accurate than those did not. In their study, a Bayesian functional regression analysis using all hyperspectral bands was integrated in order to address the high dimensionality of hyperspectral data. Krause *et al*.^17^ used genomic marker-, pedigree-, and hyperspectral reflectance-derived relationship matrices to construct genomic-enabled BLUP models to evaluate the genetic main effects (G) and GE interactions across environments in a wheat breeding program and showed the highest prediction accuracies when combining marker/pedigree information with hyperspectral reflectance phenotypes.

Physiological traits including chlorophyll content (SPAD-based), canopy temperature (CT), membrane thermostability (MT), and normalized difference vegetation index (NDVI) have shown significant association with grain yield in wheat, especially under stressed environments^18–23^. However, studies on prediction of grain yield using field-based physiological traits are limited. Weber *et al*.^24^ showed that, in maize, introducing spectral reflectance measurements at anthesis and milk-grain stage into a partial least square regression (PLSR) model accounted for 23% and 40% of the genotypic variation in grain yield, respectively. Their PLSR models explained a higher proportion of the genetic variation in grain yield under drought stress than that under well-watered conditions.

The prediction of grain yield could be potentially more accurate when a multi-kernel model is implemented in GS, in which multi-traits data and dense molecular marker information are converted into a set of distance matrices and formulated in a semi-parametric Reproducing Kernel Hilbert Space^25,26^. Pérez *et al*.^27^ applied a Bayesian-based prediction model utilizing both molecular markers and pedigree information and extended it to a multi-kernel prediction model suitable for combining multiple omic data^28^. This approach was proved to increase prediction accuracies in maize and wheat^14,16,17,29^. Therefore, the objectives of this study were to: 1) propose models using genomic and field-based physiological data to predict the grain yield in a soft facultative wheat panel, 2) compare the prediction accuracies of the model that combined field-based physiological traits with genomic data under a multi-environment context to the model that was built on either physiological traits or genomic data, and 3) rank the importance of contribution by different physiological traits to grain yield.

## Results

### Location and weather

**Figure 1:**
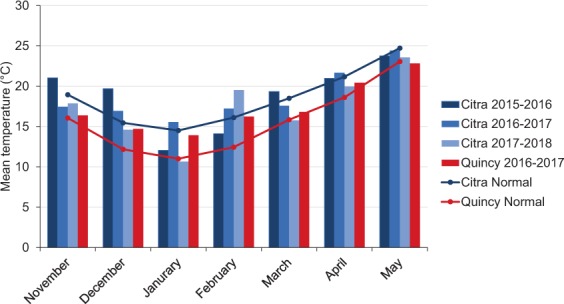
Monthly mean air temperature (60 cm; °C) during growing season 2015, 2016, 2017, and 2018 (November-May) with 30-year average at Citra, FL and Quincy, FL. (Mean temperature was from Florida Automated Weather Network and National Weather Service (FAWN), accessed on June 1^st^, 2019; 30-year monthly average was from NOAA National Centers for Environmental Information, accessed on June 1^st^, 2019).

**Figure 2:**
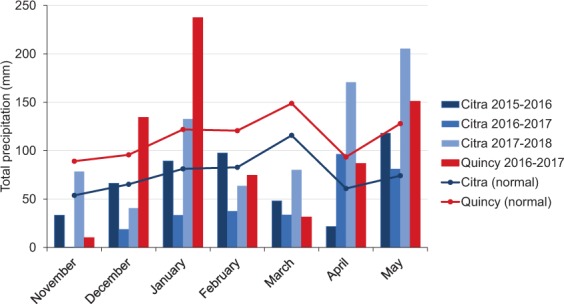
Monthly total precipitation (mm) during growing season 2015, 2016, 2017, and 2018 (November-May) with 30-year average at Citra, FL and Quincy, FL. (Mean temperature was from Florida Automated Weather Network and National Weather Service (FAWN), accessed on June 1^st^, 2019; 30-year monthly average was from NOAA National Centers for Environmental Information, accessed on June 1^st^, 2019).

In general, Quincy had lower temperatures than Citra from November of first year to May of second year (Fig. 1). However, the differences in temperature were smaller from March to May compared to other months. Unusually high precipitation occurred during January in Quincy 2017, and April and May in Citra 2018. Low precipitation was observed in March at both locations throughout the experimental period (Fig. 2). Additionally, the two locations had different soil types, where Citra had a sandy soil profile compared to a heavier soil texture in Quincy.

### Descriptive statistics

**Figure 3:**
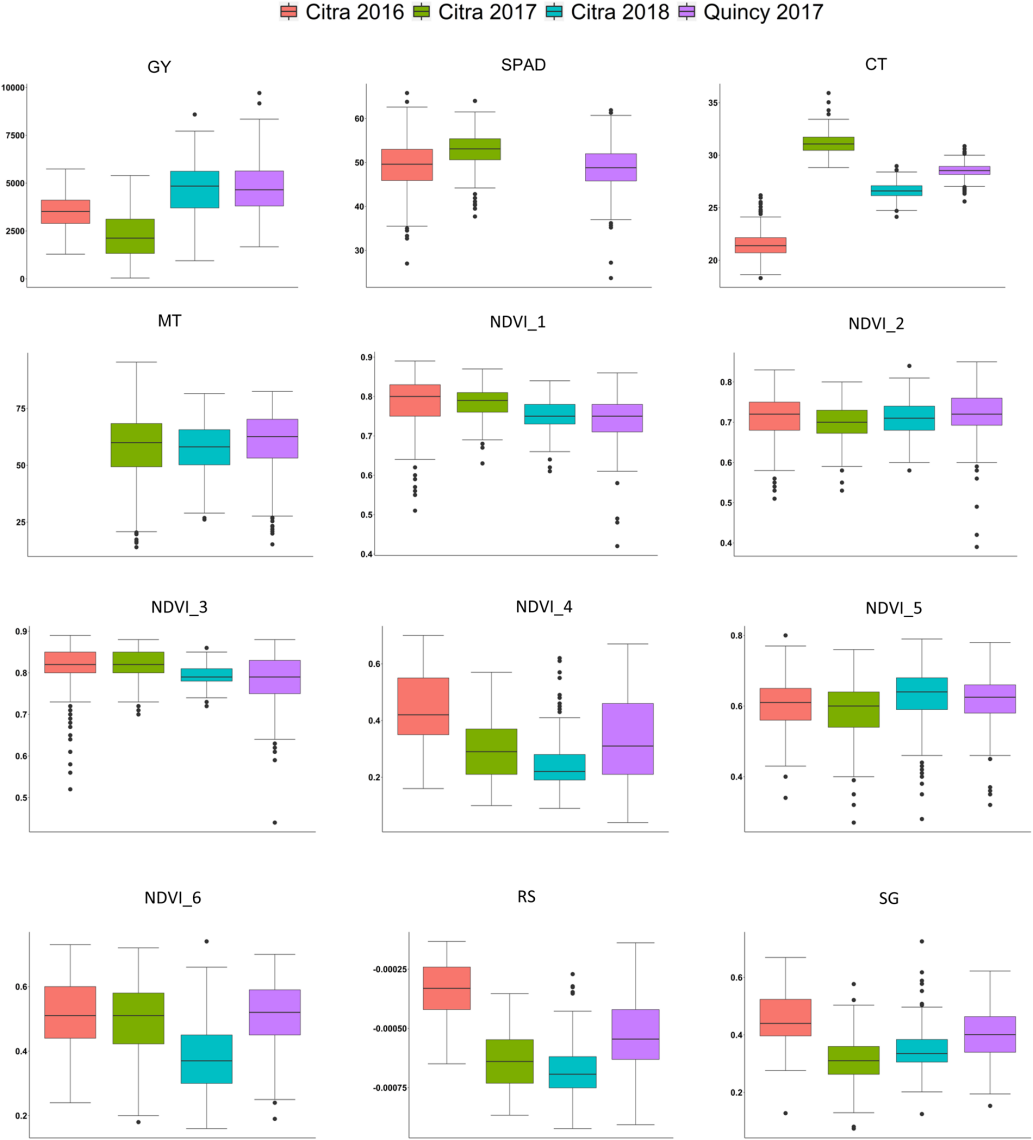
Box plots of least squares means for grain yield and 11 physiological traits evaluated at Citra, FL in 2016, 2017, and 2018, and Quincy, FL in 2017.

The same soft wheat panel was evaluated at four environments: Citra 2016, Citra 2017, Citra 2018, and Quincy 2017. Quincy 2017 and Citra 2018 showed higher GY and earlier days to heading (DTH) than Citra 2016 and 2017 (Table 1, Fig. 3). The SPAD and MT data were not taken in Citra 2018 and Citra 2016, respectively. The LSmeans of SPAD, MT, and NDVI measured at six time points were similar among environments. In Citra 2016, CT was the lowest compared to other environments. For RS, Citra 2018 had the lowest value while Citra 2016 had the highest value. In Citra 2016, SG value was highest compared to other environments. Overall, physiological traits in Citra 2016 showed higher variability than other environments (Figs. 3, S1). Broad sense heritability estimates for GY were between 0.2 and 0.41. For DTH, heritabilities were generally high between 0.69 and 0.95. Quincy 2017 showed the lowest heritability (0.24) while Citra 2016 was the highest (0.74) for SPAD. Heritabilities of MT highly varied among environments from 0.19 to 0.75. The NDVI values showed much lower heritability in Citra 2016 than other environments especially for NDVI_1, NDVI_2, NDVI_3, and NDVI_4. For RS, the heritabilities ranged from 0.43 to 0.87 among environments. Heritabilities of SG varied from 0.14 to 0.96 among environments.

**Table 1:** Description of grain yield, days to heading, and 11 physiological traits^†^ evaluated at Citra, FL in 2016, 2017, and 2018, and Quincy, FL in 2017.

Trait	Citra 2016				Citra 2017				Citra 2018				Quincy 2017			
	Mean	SE	*H*^*2*^	*p*^§^	Mean	SE	*H*^*2*^	*p*	Mean	SE	*H*^*2*^	*p*	Mean	SE	*H*^*2*^	*p*
GY^†^	3500ab^‡^	802	0.26	—	2257b	555	0.41	—	4673a	847	0.36	—	4776a	1279	0.20	—
DTH	110	1.8	0.95	−0.13	111	4.3	0.91	−0.71***	105	3.1	0.69	−0.5***	102	3.7	0.82	−0.05
SPAD	49.5	4.3	0.74	0.38***	52.7	3.0	0.53	0.14*	—	—	—	—	48.6	4.5	0.24	0.27***
CT	21.4b	1.0	0.36	−0.07	31.1a	0.9	0.04	−0.29***	26.61ab	0.6	0.12	−0.33***	28.5a	0.6	0.26	−0.24***
MT	—	—	—	—	56.4	9.5	0.69	0.31***	57.13	12.24	0.19	0.04	59.34	8.17	0.75	0.2**
NDVI_1	0.81	0.04	0.13	−0.22**	0.82	0.03	0.62	−0.35***	0.79	0.02	0.67	−0.26***	0.78	0.04	0.60	0.06
NDVI_2	0.78	0.04	0.33	−0.23***	0.78	0.02	0.80	−0.45***	0.75	0.03	0.58	−0.17**	0.74	0.04	0.68	0.12
NDVI_3	0.71	0.03	0.09	−0.23***	0.7	0.03	0.57	0.2**	0.71	0.02	0.74	−0.26***	0.72	0.04	0.63	0.14*
NDVI_4	0.6	0.03	0.32	−0.13	0.58	0.06	0.52	−0.09	0.62	0.08	0.38	−0.000739458	0.62	0.06	0.51	0.12
NDVI_5	0.51a	0.03	0.80	−0.07	0.5a	0.05	0.73	−0.43***	0.38b	0.08	0.34	−0.29***	0.51a	0.07	0.66	0.05
NDVI_6	0.44a	0.04	0.90	−0.07	0.29b	0.08	0.34	−0.42***	0.25b	0.06	0.39	−0.44***	0.34ab	0.09	0.79	−0.06
RS	−0.00034b	0.00005	0.87	0.06	−0.00064a	0.00008	0.43	−0.44***	−0.00068a	0.00007	0.78	−0.4***	−0.00053a	0.00009	0.62	−0.10
SG	0.45a	0.03	0.96	−0.02	0.31b	0.05	0.46	0.18**	0.35ab	0.06	0.14	−0.27***	0.4a	0.07	0.35	0.09

*Significant at the *P* < 0.05, **Significant at the *P* < 0.01, ***Significant at the *P* < 0.001.

^†^GY = Grain Yield; DTH = Days to Heading; SPAD = SPAD Chlorophyll Content; CT = Canopy Temperature; MT = Membrane Thermostability; NDVI = Normalized Difference Vegetation Index; RS = Rate of Senescence; SG = Stay Green.

^‡^Values with different letters are significantly different by Tukey’s studentized range test at 0.05 level of probability, no letter was assigned if values were not significantly different.

^§^Pearson correlation coefficient between GY and physiological traits.

Correlations between physiological traits and GY also highly varied among environments (Table 1). Heading date significantly correlated with GY in Citra 2017 and Citra 2018 but was not correlated with GY in Citra 2016 and Quincy 2017. In the three environments, SPAD were positively correlated with GY (*p* = 0.38, 0.14, 0.27 at Citra 2016, Citra 2017, and Quincy 2017, respectively). In Citra 2017 and Quincy 2017, MT values were positively correlated with GY (*p* = 0.31 and 0.2 at Citra 2017 and Quincy 2017, respectively). The first three NDVI measurements (NDVI_1, NDVI_2, and NDVI_3) were negatively correlated with GY (*p* from −0.45 to −0.17) except for Quincy 2017 (*p* from 0.06 to 0.14). NDVI_5 and NDVI_6 were negatively correlated with GY in Citra 2017 and Citra 2018 (*p* from −0.43 to −0.42 and −0.44 to −0.29 at Citra 2017 and Citra 2018, respectively). Citra 2017 and Citra 2018 showed negative correlations between RS and GY (*p* = −0.44 and −0.4 at Citra 2017 and Citra 2018, respectively). For SG, Citra 2017 showed positive correlation with GY and Citra 2018 had negative correlation (*p* = 0.18 and −0.27 at Citra 2017 and Citra 2018, respectively).

### Model prediction accuracy

**Figure 4:**
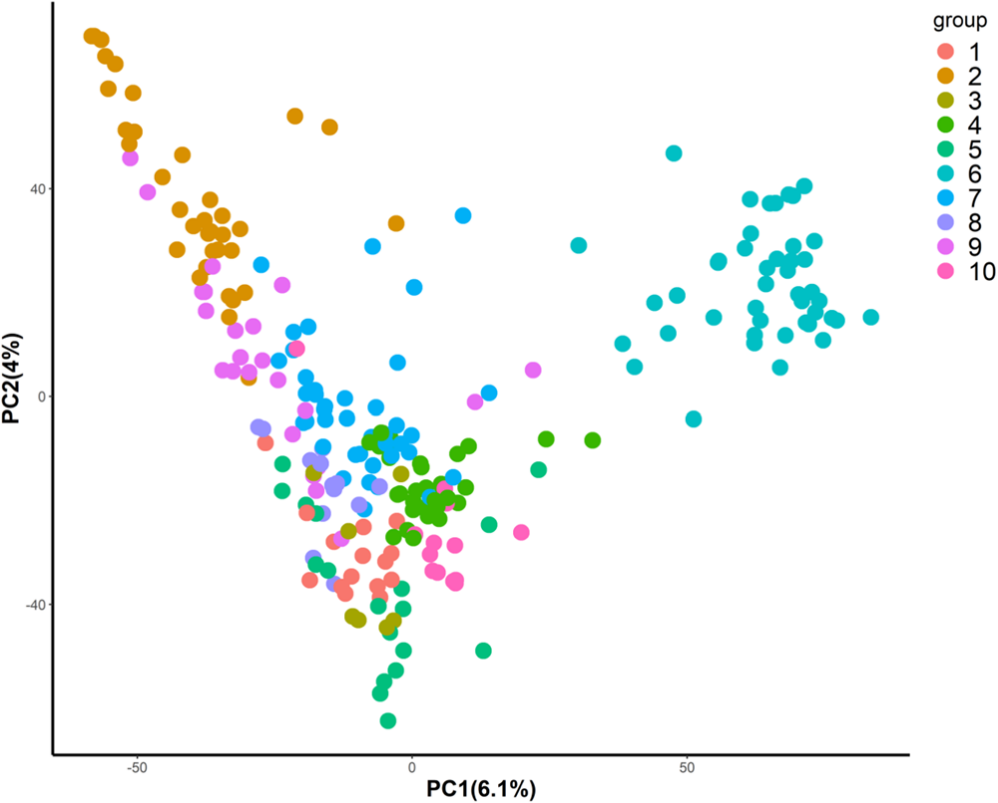
Stratification of genomic selection panel inferred from discriminant analysis of principal components (DAPC) using 19,353 SNPs data.

**Figure 5:**
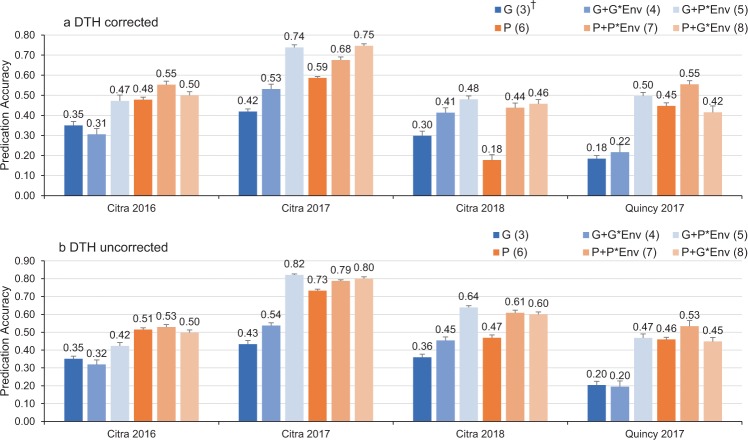
Prediction accuracies for grain yield using six models, with and without correction for DTH.

To determine the population structure in the soft wheat panel, 242 lines were clustered into 10 groups using the *DAPC* algorithm (Fig. 4). Each sub-group consisted of 21 to 35 lines which were then randomly assigned to five different folds for cross validation analysis. When DTH was used as a covariate, prediction accuracies of models using model (3) (***G*** only) ranged from 0.18 (Quincy 2017) to 0.42 (Citra 2017) (Fig. 5a). When model (6) (***P*** only) was used, all environments showed higher prediction accuracies (from 0.18 to 0.59) than model (3) except for Citra 2018. Model (4) including ***G*** and ***G*** × ***E*** interaction showed higher prediction accuracies than model (3) except for Citra 2016. Model (5) including ***G*** and ***P*** × ***E*** showed the highest prediction accuracies in Citra 2018 (0.48) among six models. Model (7) using ***P*** and ***P*** × ***E*** had the highest prediction accuracies in Citra 2016 (0.55) and Quincy 2017 (0.55). Model (8) using ***P*** and ***G*** × ***E*** had the highest prediction accuracy in Citra 2017 (0.75). When DTH was not used a covariate, all environments showed similar patterns as models with DTH corrected (Fig. 5b). However, the prediction accuracies increased across environments except for models with ***G*** or ***G*** + ***G*** × ***E***. Whether DTH being corrected or not, models incorporating physiological traits (***P***) and environmental effects (***E***) performed better than ***G*** or ***P*** only, with exceptions when comparing model (6) and model (8) at Quincy 2017 and Citra 2016.

### Response to selection

**Figure 6:**
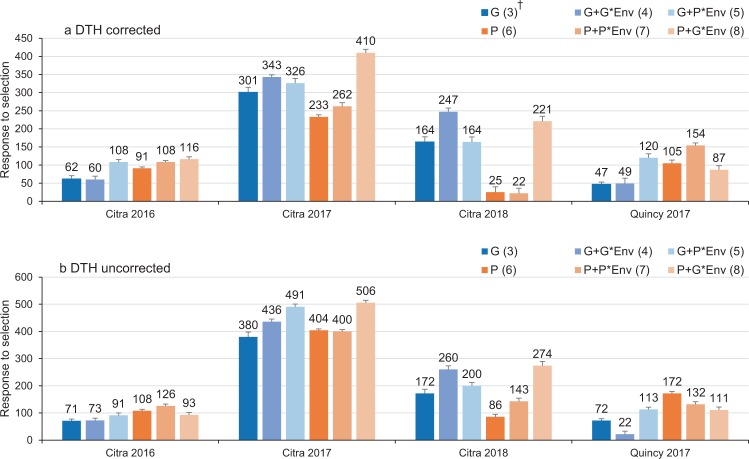
Response to selection for grain yield using six models, with and without correction for DTH.

When comparing the response to selection (RTS) based on each model in each environment, models considering both ***P*** and ***G*** × ***E*** showed generally higher performance (116, 410, and 221 at Citra 2016, Citra 2017, and Citra 2018, respectively) with DTH correction (Fig. 6) than other models. In environments where GY showed relatively low heritability (i.e. Citra 2016 and Quincy 2017), models involved with ***P*** or ***P*** × ***E*** had higher RTS compared to models with ***G*** or ***G*** × ***E***. On the contrary, in environments with higher heritability for GY (i.e. Citra 2017 and Citra 2018), models included ***G*** or ***G*** × ***E*** showed higher RTS than models included ***P*** or ***P*** × ***E***. In Citra 2018, models of ***P*** and ***P*** + ***P*** × ***E*** had the lowest RS (25 and 22, respectively) among all environments. When DTH was not used as the covariate for GY, a similar pattern but generally higher RTS was observed for all the environments (Fig. 6b). Models (8) which involving ***P*** and ***G*** × ***E*** showed the highest RTS at Citra 2017 and Citra 2018 (506 and 274, respectively), followed by model (5) involving ***G*** and ***P*** × ***E*** (491 and 260, respectively). Model (4) at Quincy 2017 and model (6) at Citra 2018, formulated as ***G*** + ***G*** × ***E*** and ***P***, respectively, had the lowest RTS (22 and 86, respectively) across all environments.

### Multi-variate analysis for physiological traits

**Figure 7:**
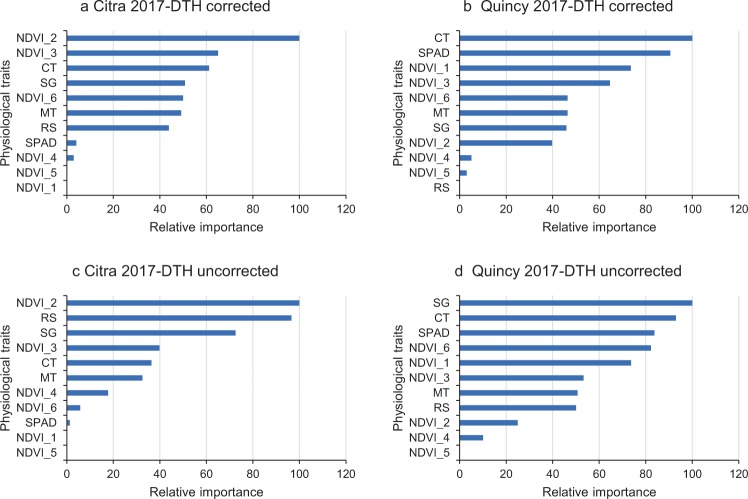
Rank of importance of physiological traits in predicting grain yield using machine learning based clustering analysis.

The relative importance of physiological traits contributing to GY was analyzed using machine learning based clustering method. Data from Citra 2017 and Quincy 2017 was selected for this analysis since all physiological traits were available in these two environments. The cross-validation results (alpha = 1; lambda = 10.3) suggested using a least absolute shrinkage and selection operator (LASSO) model to estimate the coefficients of each variables. When DTH was not included as a covariate, the dominant contributor to GY in Citra 2017 was NDVI_2 followed by NDVI_3 and CT. The relative contribution of top three traits was scored as 100, 65, and 61, respectively (Fig. 7a). For Quincy 2017, CT was the dominant contributor to GY followed by SPAD (90), NDVI_1 (74), and NDVI_3 (64) (Fig. 7b). In both environments, NDVI_4 and NDVI_5 were not very important. When DTH was not included as a covariate, the overall rank of physiological traits was similar in each environment compared to analysis with DTH (Figs. 7c,d). In Citra 2017, RS became the second most important contributor to GY when DTH was included. In Quincy 2017, SG rose to be the most important contributor to GY, and RS also ranked higher compared to analysis with DTH included.

## Discussion

### Model prediction accuracy

Our study found that, considering both field-based physiological measurements, genomic information, and genotype × environment (or physiology × environment) in a multi-kernel BLUP model can significantly improve prediction accuracy for GY. Physiological traits such as SPAD, CT, MT, and NDVI measured between heading and maturity stage are reported to be effective to predict GY^18–23^. When physiological traits were added to multi-kernel models (5, 7, and 8), the prediction accuracies were similar to previous reports^17,30^. In addition, the prediction accuracies in the current study are also site-specific in terms of magnitude of differences between ***G*** and ***G*** + ***P*** models. However, in their studies, thermo- and spectral camera-based imaging analysis was implemented to measure CT and vegetation indices which had a higher heritability than the physiological traits measured in this study. Prediction models using a multi-variate set of phenotypic data has proven to enhance the prediction accuracy compared to using a single phenotypic trait^16,17^. In our study, the prediction accuracies using only ***P*** matrices are close to other models in several environments. The addition of genomic information and interactions in models improved prediction accuracies only in a marginal scale in those environments. The missing SPAD measurement in Citra 2018 could contribute to a much lower prediction accuracy using only ***P*** matrix. This result is consistent to Krause *et al*.^17^ and Montesinos-López *et al*.^11^.

In this study, when DTH is corrected in estimating GY, prediction accuracies for the models involving only ***G*** matrices were similar to models without DTH correction. However, when ***P*** matrices were included in these models, prediction accuracies were generally reduced in all environments except for Citra 2016 and model (7) in Quincy 2017, which agrees with Krause *et al*.^17^ and Rutkoski *et al*.^30^. However, the magnitudes of differences between models with and without DTH correction were marginal except for Citra 2018. This is probably due to missing data on SPAD in Citra 2018. This again indicated that SPAD plays an important role in contributing to GY. Correcting GY for DTH can avoid indirect selection on maturity traits. Our results confirmed that variation in physiological traits such as NDVI values is correlated with maturity differences among lines. Thus, the prediction accuracies derived after DTH correction are more informative.

### Response to selection

For a plant breeding program, it is a common practice to evaluate the genetic gain based on response to selection^31^. In this study, we calculated RTS based on GEBV from the proposed models for each environment. In general, environments with higher heritability for GY had higher RTS. Model (8) (***P*** and ***G*** × ***E***) and model (4) (***G*** and ***G*** × ***E***) showed to be superior than other models in environments where GY had high heritability. However, in a low heritability environment such as Citra 2016 or Quincy 2017 in our study, selection using physiological traits (***P*** or ***P*** × ***E***) could perform better than GS (***G*** or ***G*** × ***E***). Crain *et al*.^15^ also found that physiological traits can be used to improve model performance over GS models alone in different environments. However, high-throughput phenotyping techniques were used to measure physiological traits such as NDVI and CT in their study. Ultimately, based on our results, the potential of increasing genetic gains for GY can be achieved by applying a multi-kernel model implemented with information of physiological traits.

### Multi-variate analysis for physiological traits

Because the same set of physiological traits were measured in Citra 2017 and Quincy 2017, data from these two environments were used to investigate the importance of physiological traits contributing to GY. The machine learning-based clustering analysis indicated that importance of physiological traits to GY are environment specific. However, NDVI_3 that was measured at the end of milk stage (Zadoks 79) and CT were ranked on the top in both environments. Previous studies showed milk-grain or milk-dough stages were critical in determining the grain yield in various environments^32–34^. High throughput phenotyping methods such as satellite imaging, UAV spectral imaging and proximal phenotyping are advancing at a rapid pace^35–38^. It is now possible to collect vegetative indices across all growth stages. The results from the clustering analysis suggest that, phenotyping plants during milk-grain stages could improve the prediction accuracy on GY. The impact of canopy temperature on grain yield was also well documented^19,35,39^. Cooler CT is associated with higher stomatal conductance and better hydration status under drought condition, in return it results in a higher yield^40,41^. Therefore, using thermo-camera to collect CT data during grain filling stage could also improve the prediction accuracy on GY. For Quincy 2017, SPAD was the top contributor to GY. In previous studies, higher chlorophyll content was associated with greater grain yield especially under drought conditions^42–44^. There was a drought period during March 2017, which could result in a lower yield for the genotypes that were vulnerable to the drought conditions. Therefore, SPAD measurement played a more important role in Quincy 2017 than that in Citra 2017. When GY was not corrected for DTH, the overall pattern remained the same for each environment except for a higher rank of RS and SG on both lists. Since RS and SG are directly correlated with NDVI values and maturity, they would be inherently correlated with phenology. Therefore, the attention on RS and SG should be weighed carefully when a diverse panel of germplasm is evaluated.

## Conclusions

In this study, we combined field-based phenotyping and genomic information to predict grain yield using multi-kernel models in the soft wheat panel. The multi-kernel models and single-kernel model using physiological trait information provided better prediction accuracies than the single-kernel model using genomic data only. Therefore, applying high throughput phenotyping on SPAD, CT, MT, and NDVI during milk-grain stages could potentially accelerate selection and advancing germplasm in wheat. The multi-dimensional aerial- or ground-based high throughout phenotyping information could in turn argument the selection of traits used to predict GY. Although the classification of time-point when these traits are collected is probably specific to environments and genetic backgrounds of the lines, the importance of certain period during growth stages could be easily evaluated using the same methodology proposed in this study. Our study provides baseline information on using physiological traits to predict grain yield in wheat in a multi-environment context.

## Materials and Methods

### Plant materials and experimental design

A diversity panel of 242 soft facultative wheat with relatively low vernalization requirement for most of the genotypes was used in the present study. These lines were released from public and private soft wheat breeding programs in the southern and southeastern U.S. to represents a broad genetic base of US soft wheat. The panel was phenotyped for both physiological and yield related traits at the Plant Science Research and Education Unit (PSREU) in Citra, Florida from 2016 to 2018 and at the North Florida Research and Education Center (NFREC) in Quincy, Florida in 2017. All field experiments were planted in an un-replicated randomized augmented design with three repeated checks (“AGS 2000”, “SS8641”, and “Jamestown”). Each line was planted in six row plots (3 m × 1.5 m) at the rate of 100 kg h^-1^. Pesticides were sprayed for management of local diseases, weeds, and insects as needed. Fertilizer and irrigation were applied based on plant growth stages and field moisture condition to avoid any water or nutrient deficiency, respectively. Planting dates were delayed to late December to increase post-anthesis heat stress conditions. Weather data, including average temperature (60 cm above canopy) and precipitation, were retrieved from Florida Automated Weather Network (FAWN) and the National Oceanic and Atmosphere Administration (NOAA) for each environment (Figs. 1 and 2).

### Field data collection and calculations

Physiological traits including SPAD, CT, MT, NDVI values at six time points (Zadoks stages 65, 72, 79, 86, 93 and 100, respectively), RS, and SG were measured from each plot in each experiment. SPAD chlorophyll content of flag leaf was measured when plants reached early milk stage (Zadoks stage 72, or seven days after anthesis) using a handheld chlorophyll meter (Minolta SPAD-502 Spectrum Technologies Inc., Plainfield, IL, US). CT was recorded three times during grain filling using a handheld infrared thermometer (Fluke 572-2 IR thermometer, Fluke Corporation, Everett WA) during cloudless, sunny day when the temperature reached daily high. To determine MT, flag leaves were collected from ten random main stem at early milk stage (Zadoks stage 72). One-centimeter diameter leaf disks were collected from the middle section of the ten leaf blades using a paper puncher and placed in glass vial containing 20 ml deionized water. The leaf samples were then processed following Ibrahim & Quick^45^ and MT was expressed in percentage units as the reciprocal of relative electrolyte leakage measured by conductometer (Thermo Scientific Orion Star A212) followed by autoclaving the vials (0.10 MPa pressure, 121 °C for 15 min) to release all the electrolytes from plant tissue.

$${\rm{MT}}=(1\mbox{--}{{\rm{T}}}_{1}/{{\rm{T}}}_{2})\times 100$$

where T_1_ is the conductivity reading after heat treatment, and T_2_ is the conductivity reading after autoclaving. All NDVI values were measured using a GreenSeeker sensor (Trimble Navigation, Ltd., Sunny Vale, CA, USA). The GreenSeeker was held 30 cm above the canopy and scan through the center of each plot. An averaged reading was recorded for each plot in each environment. Rate of senescence was calculated as the slope of the linear NDVI decline over accumulated growing degree days (AGDD) based on Harris *et al*.^46^ and Lopes & Reynolds^47^. Stay green score was estimated using the predicted NDVI value at physiological maturity according to Lopes & Reynolds^47^. Specifically, the linear regression equation obtained from the NDVI decay during grain-filling against accumulated AGDD after heading was first generated, and then days to physiological maturity was introduced into the equation to calculate the corresponding NDVI value.

$${\rm{ND}}\widehat{{{\rm{VI}}}_{{\rm{AGDD}}}}=m{\rm{AGDD}}+b$$

$${{\rm{NDVI}}}_{{\rm{PM}}}=m{{\rm{AGDD}}}_{{\rm{PM}}}+b$$

where NDVI_AGDD_ is the simulated NDVI value at AGDD (°C days), NDVI_PM_ (i.e. SG value) is the predicted NDVI value of AGDD at physiological maturity, AGDD_PM_ is the AGDD at physiological maturity, *m* and *b* is the slope and intercept of the linear regression model, respectively. Grain yield was calculated by dividing total grain weight from each plot by the plot area, adjusted to 12% moisture level and expressed in kg ha^-1^. Heading date was recorded as the number of days from planting date to the day when 50% spikes emerged in each plot.

### Genotypic data analysis

High quality DNA was isolated from freeze-dried, powdered leaf tissue (~100 mg) collected from two-week-old plants using a modified cetyltrimethylammonium bromide protocol^48,49^. The genotyping-by-sequencing (GBS) libraries were prepared using *MspI* and *PstI-*HF restriction enzymes^50^. The libraries were pooled together in 96-plex and sequenced in an Ion Torrent Proton sequencer (Thermo Fisher Scientific, Waltham, MA, USA) following manufacturer’s instructions at the USDA Central Small Grain Genotyping Lab, Kansas State University, Manhattan, KS, USA. All 242 soft wheat lines were genetically characterized using GBS approach^51^.

SNP calling was performed using the TASSEL v5.0 GBS v2.0 discovery pipeline^52^. From the initial set of 448,307 sites, 49,406 SNPs remained after filtering markers with more than 80% of missing data and minor allele frequency less than 0.05. Missing values were imputed with LD-KNNi method^53^ implemented in TASSEL v.5. A Fisher exact test was used to test if the SNP alleles were independent in a population of inbred lines as described by Poland *et al*.^54^. The SNPs were assumed allelic in the population if the null hypothesis of independence for the two alleles was rejected (*P* < 0.001). This procedure typically lowers heterozygous calls due to sequencing errors, genome duplications, and homologous sequences on different genomes^50,54,55^. In the final genomic dataset, a total of 19,353 SNPs remained.

### Phenotypic data analysis

Least squares mean (LSmean) and standard error of grain yield and physiological traits for each environment were obtained using the following model with genotype as a fixed effect and location and block as random effects:

1 $${Y}{{\prime} }_{{ijk}}=\mu +G{g}_{j}+{E}_{i}+{B}_{i(k)}+Gg{E}_{ji}+{e}_{ijk}$$

where *Y*_*ijk*_ is the observed value; µ was the general genotype mean; $G{g}_{j}$ is the genotypic effect (j = 1 to 242); ${E}_{i}$ is the environment effect (i = 1 to 4, corresponding to Citra 2016, Citra 2017, Citra 2018, and Quincy 2017); ${B}_{j(k)}$ is the block effect (k = 1 to 12) within the i^th^ environment; $Gg{E}_{ji}$ is the j^th^ genotype by i^th^ environment interaction effect; and ${e}_{ijk}$ is the random error. To evaluate the influence of phenology, DTH was included as a covariate in model (1) when calculating LSmeans (i.e. corrected GY). Therefore, two sets of data including LSmeans of corrected and uncorrected GY were used for all following analyses, separately. To calculate broad sense heritability (*H*^2^) of grain yield and physiological traits for each environment, the following model was used to obtain variance of each effect:

2 $${Y}{{\prime} }_{{ij}}=\mu +G{g}_{j}+{B}_{k}+{e}_{jk}$$

In this model, genotype and block were considered as random effects. Broad sense heritability (*H*^2^) from each environment was calculated using the following formula, H^2^ = (σ^2^_G_)/(σ^2^_G_+σ^2^_e_), where, σ^2^_G_ and σ^2^_e_ were variances due to genotype and error, respectively. LSmeans were for all traits at each location and used for Pearson correlation analyses between grain yield and other traits. Difference in LSmeans for all the traits among environments was claimed to be significant at *P* = 0.05 using Tukey’s Post-Hoc test.

### Prediction models

According to Montesinos-López *et al*.^16^, Krause *et al*.^17^ and Jarquín *et al*.^56^, six BLUP of models incorporated with combinations of marker information (***G***), physiological data (***P***), marker × environment (***G*** × ***E***), and physiological data × environment (***P*** × ***E***) were proposed to assess the prediction accuracy of grain yield. Environmental effect was considered as a fixed effect in all models. Accordingly, the following models, models (3) to (8), were fitted as ***G*** only, ***G*** + ***G*** × ***E***, ***G*** + ***P*** × ***E***, ***P*** only, ***P*** + ***P*** × ***E***, and ***P*** + ***G*** × ***E***, respectively:

3 $${\rm{G}}:{Y}_{ij}=\mu +{E}_{i}+{G}_{j}+{\varepsilon }_{ij}$$

4 $${\rm{G}}+{{\rm{G}}}^{\ast }{\rm{Env}}:{Y}_{ij}=\mu +{E}_{i}+{G}_{j}+G{E}_{ij}+{\varepsilon }_{ij}$$

5 $${\rm{G}}+{{\rm{P}}}^{\ast }{\rm{Env}}:{Y}_{ij}=\mu +{E}_{i}+{G}_{j}+P{E}_{li}+{\varepsilon }_{ij}$$

6 $${\rm{P}}:{Y}_{ij}=\mu +{E}_{i}+{P}_{l}+{\varepsilon }_{il}$$

7 $${\rm{P}}+{{\rm{P}}}^{\ast }{\rm{Env}}:{Y}_{ij}=\mu +{E}_{i}+{P}_{l}+\ast P{E}_{li}+{\varepsilon }_{il}$$

8 $${\rm{P}}+{{\rm{G}}}^{\ast }{\rm{Env}}:{Y}_{ij}=\mu +{E}_{i}+{P}_{l}+G{E}_{ji}+{\varepsilon }_{il}$$

Where *Y*_*ij*_ is the LSmean of GY for *j*^th^ genotype in *i*^th^ environment; *μ* is the overall mean; ${E}_{i}$ is the environment effect (*i* = 1 to 4, corresponding to Citra 2016, Citra 2017, Citra 2018, and Quincy 2017); ${G}_{j}$ is the genetic main effect (*j* = 1 to 242); the genetic main effect is assumed as a joint distribution of genotype effect with a multivariate normal distribution $G={({G}_{1},\ldots ,{G}_{j\ast })}^{T}\sim MN(0,\,{\sigma }_{G}^{2}{\boldsymbol{G}})$, where ${\sigma }_{G}^{2}$ denotes the genomic variance and ***G*** represents the genomic relationship matrix; the ***G*** matrices were calculated as ${\boldsymbol{G}}=\frac{{\boldsymbol{X}}{\boldsymbol{X}}{\boldsymbol{{\prime} }}}{n}$, where ***X*** is a matrix of the centered and standardized SNP marker matrix and *n* is the number of SNP markers; $G{E}_{ji}$ is the *j*^*t*h^ genotype by *i*^th^ environment interaction effect; The term $G{E}_{ji}$ was assumed to have a multivariate normal distribution, that is $G{E}_{ji}={(G{E}_{11},\ldots ,G{E}_{ji})}^{T} \sim MN(0,\,({Z}_{g}G{Z}_{g}^{T})\#({Z}_{E}{G}_{E}^{T}({\sigma }_{GE}^{2})$ where ${Z}_{g}$ and ${Z}_{E}$ are incidence matrices for the vector of genomics and environment effects, and ${\sigma }_{GE}^{2}$ is the variance component for $G{E}_{ji}$; ${P}_{l}$ is the physiological main effect for genotype *l* with the joint distribution of six physiological traits as $P={({P}_{1},\ldots ,{P}_{l\ast })}^{T}\sim MN(0,\,{\sigma }_{P}^{2}{\boldsymbol{P}})$; where ${\sigma }_{P}^{2}$ denotes the physiological trait variance and ***P*** represents the physiological trait-derived relationship matrix, the ***P*** matrices were calculated as ${\boldsymbol{P}}=\frac{{\boldsymbol{S}}{\boldsymbol{S}}{\boldsymbol{{\prime} }}}{m}$, where ***S*** is a matrix of the centered and standardized LSmeans of six physiological traits and *m* is the number of physiological variables; $P{E}_{li}$ is the physiological matrices of *l*^*t*h^ genotype by *i*^th^ environment interaction effect; and ${\varepsilon }_{il}$ was the random error. The term $P{E}_{li}$ was assumed to have a multivariate normal distribution, that is $P{E}_{li}={(P{E}_{11},\ldots ,P{E}_{li})}^{T}\sim MN(0,\,({Z}_{g}P{Z}_{g}^{T})\#({Z}_{E}{Z}_{E}^{T}){\sigma }_{PE}^{2})$ where ${Z}_{g}$ and ${Z}_{E}$ are incidence matrices for the vector of genomics and environment effects, and ${\sigma }_{PE}^{2}$ is the variance component for $P{E}_{li}$ (Krause *et al*.; Jarquín *et al*. 2014).

### Model prediction accuracy

For each environment, all six models were evaluated using a five-fold cross validation approach for their prediction accuracies. Briefly, the association panel was partitioned into five equally sized (or similar) subgroups. Four of the five subgroups (i.e., the TP) were used to fit each prediction model while the remaining subgroup (i.e., the VP) was used to assess the correlation between the observed and predicted trait values. This process was repeated five times, with each subgroup being used as the prediction set for once. To control the relatedness among lines, the population was stratified based on discriminant analysis of principal components (*DAPC*)^57^ clustering analysis, so that lines belonging to the same group were present in either validation or training population, not in both simultaneously. Prediction accuracies were calculated as ${r}_{GY}={r}_{{\rm{p}}}/\sqrt{{H}^{2}}$, where ${r}_{{\rm{p}}}$ is the mean predictive correlations across five folds. Standard error of prediction accuracy for each environment and each model was calculated based on ${{\rm{SE}}}_{GYP}={{\rm{\sigma }}}_{{r}_{p}}/\sqrt{f{H}^{2}}$, where ${{\rm{\sigma }}}_{{r}_{p}}$ is the standard deviation of the predictive correlation;$\,f$ is the number of folds (5 in this case). The same procedure was performed for GY corrected for phenology (i.e. DTH included as a covariate).

In order to further evaluate the performance of prediction models, response to selection (RTS) was calculated using the formula *R* = *H*^*2*^*S*^31^, where *H*^2^ is the heritability for grain yield; and *S* is the selection differential (in unit of kg ha^−1^). In specific, all 242 lines were ordered according to their GEBV calculated from each model in each environment. The top 10% lines were then chosen as the selected population (i.e. selection intensity of 10%). Selection differential was calculated as the difference of grain yield between the means of selected lines and whole population: *S* = μ_S_ – μ_P_, where μ_S_ is the mean yield of 10% selected lines based on GEBV and μ_P_ is the mean yield of population. Response to selection for all six models at each environment were computed with and without correction for DTH. Mean of RTS was calculated for each environment and each model across five folds. Standard error of RTS was calculated based on ${{\rm{SE}}}_{GYRTS}={{\rm{\sigma }}}_{RTS}/\sqrt{f}$, where ${{\rm{\sigma }}}_{RTS}$ is the standard deviation of the RTS; $f$ is the number of folds (5 in this case).

### Multi-variate analysis for physiological traits

In order to dissect the inter-relationships between GY and physiological traits, a machine learning based clustering analysis was performed using CARET (Classification and Regression Training) technique in R. A multi-variate prediction model on GY were created using all physiological traits collected from the field. In specific, the physiological data was firstly standardized and centered before subjecting into regularized regression models that employ strict penalties to prevent overfitting. The penalty parameters control the levels of shrinkage of the coefficients for correlated predictors. In CARET, regularization path is computed for the LASSO or elastic net penalty at a grid of values for the regularization parameter alpha and lambda. A bootstrap training procedure with a 10-fold cross-validation and 20 repetitions was used to evaluate the performance of different penalty levels on GY prediction. A final model was selected based on the smallest mean squared error obtained in the training procedure. The magnitude of importance of physiological trait contributing to GY was compared based on the absolute value of scaled coefficients, with/without DTH corrected as a covariate. The dataset from Citra 2017 was used for the analysis since all 11 physiological traits were collected.

### Software

Phenotypic data analysis, including LSmeans and heritability calculation, and correlation analyses, were performed using R (R Development Core Team 2018). Basic models (1-2) were fit with the “lme4” package^58^. Prediction models (3-8) were fit with package “BGLR”^59^. The *DAPC* analysis was performed using “adegenet” package^60^. Cross-validation and prediction accuracy calculation were conducted using customized codes in R. Clustering analysis for physiological traits was performed using “caret” package^61^.

## Supplementary information

Supplementary information.

## Data Availability

All data generated or analyzed during this study are available from the corresponding author on reasonable request.
