# Supplementary material for: Increased Prediction Accuracy Using Combined Genomic Information and Physiological Traits in A Soft Wheat Panel Evaluated in Multi-Environments
Source: Sci Rep. 2020 Apr 27;10:7023. doi: 10.1038/s41598-020-63919-3 (PMC7184575; doi:10.1038/s41598-020-63919-3)
Supplement: Supplementary file 1 — Supplementary information. [file 41598_2020_63919_MOESM1_ESM.docx]

Supplemental Figure 1. Histogram of grain yield and 11 physiological traits

|  | Citra 2016 | Citra 2017 | Citra 2018 | Quincy 2017 |
| --- | --- | --- | --- | --- |
| GY | 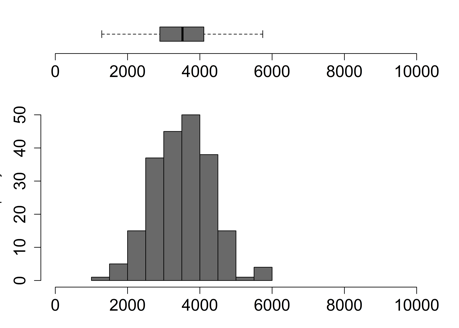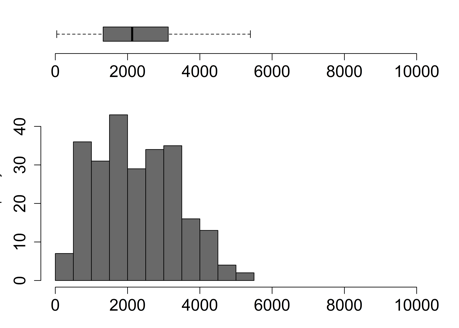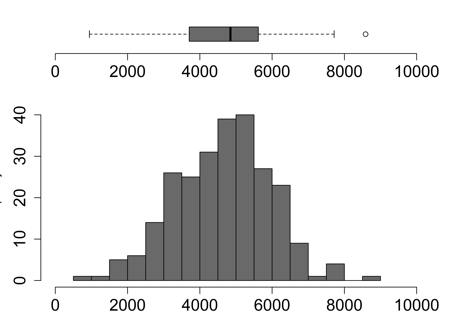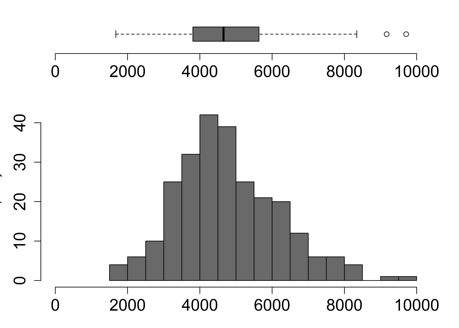 | | | |
| SPAD | 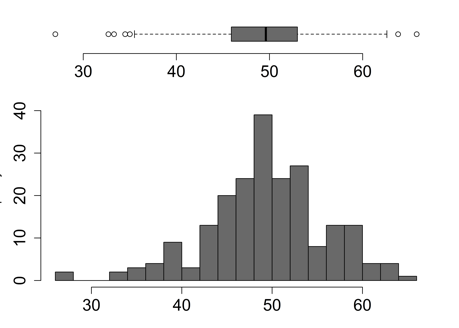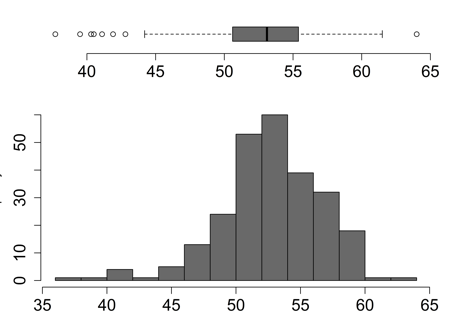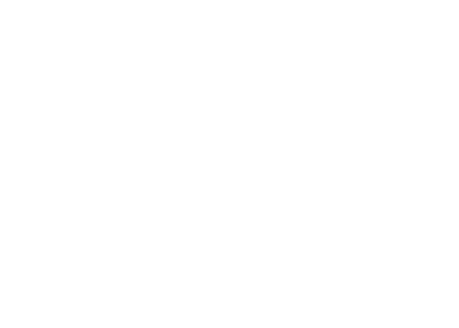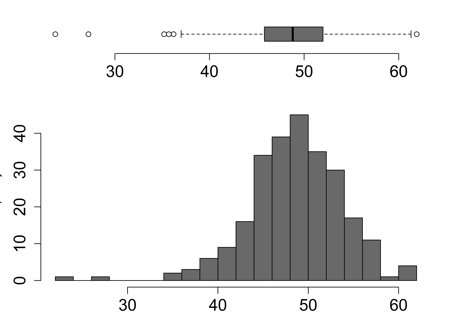 | | | |
| CT | 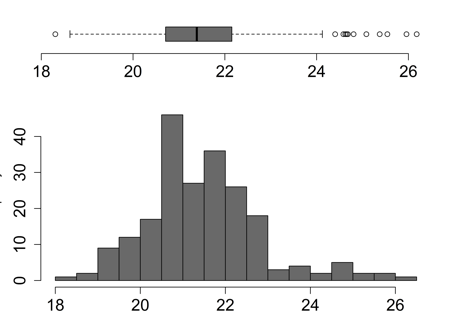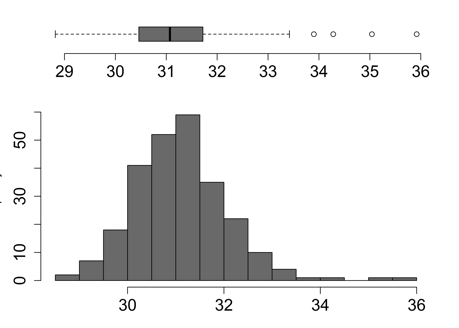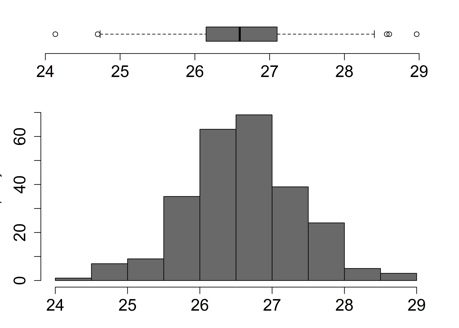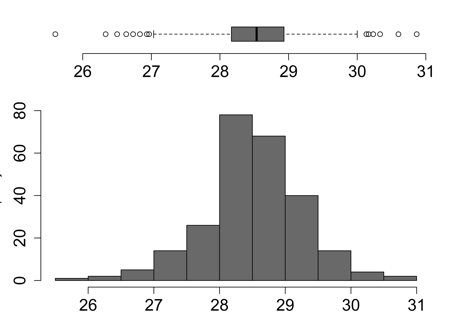 | | | |
| MT | 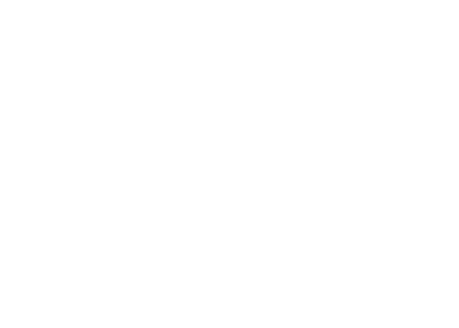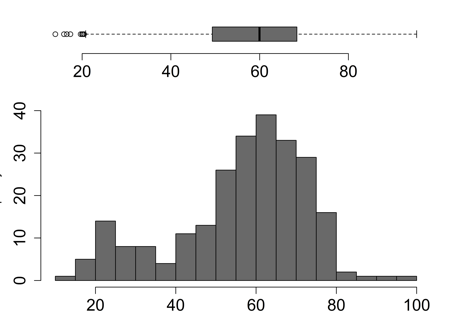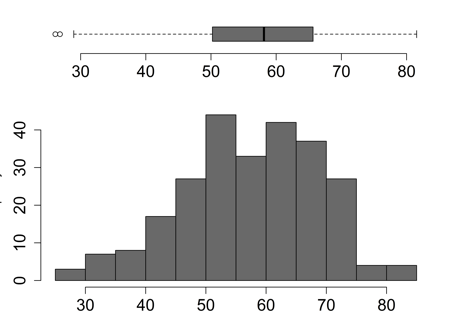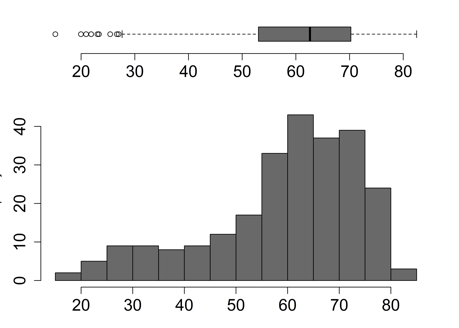 | | | |
| NDVI_1 | 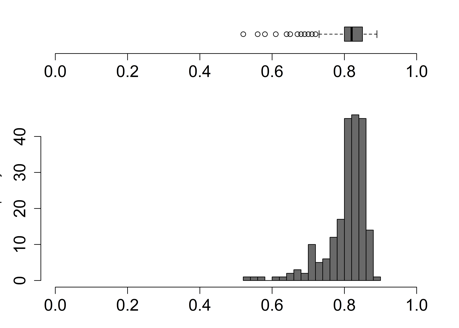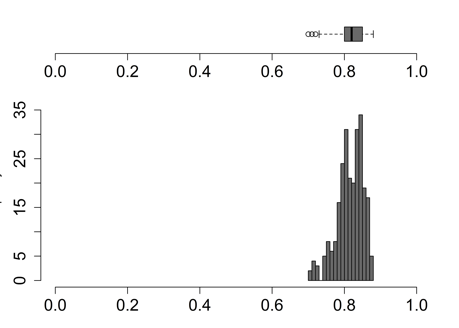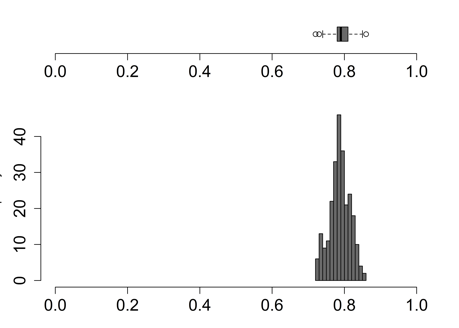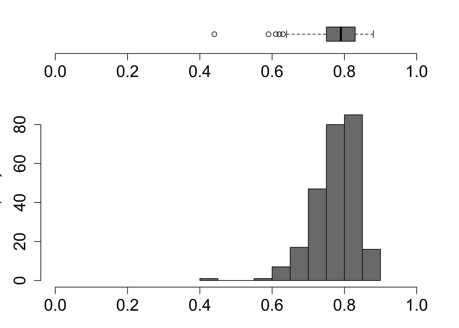 | | | |
| NDVI_2 | 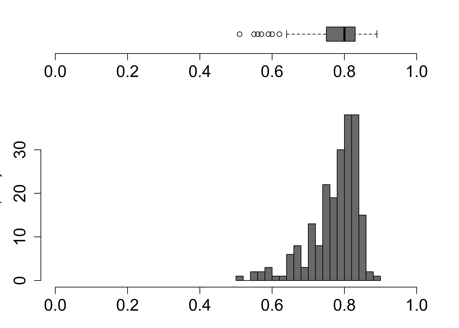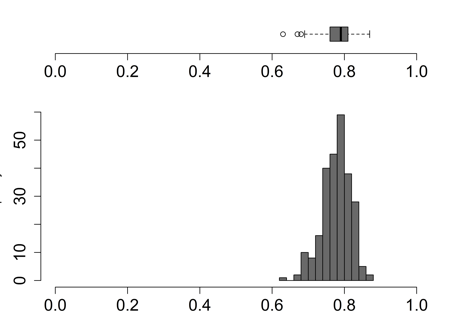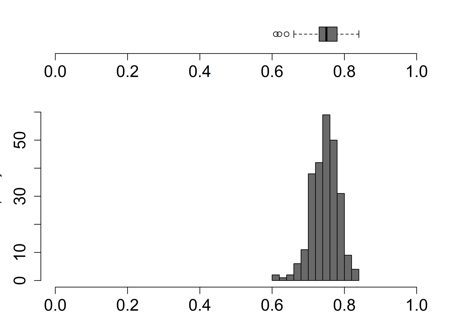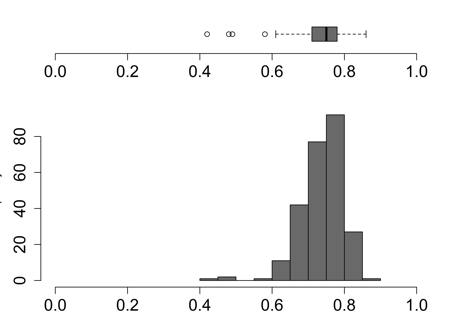 | | | |
| NDVI_3 | 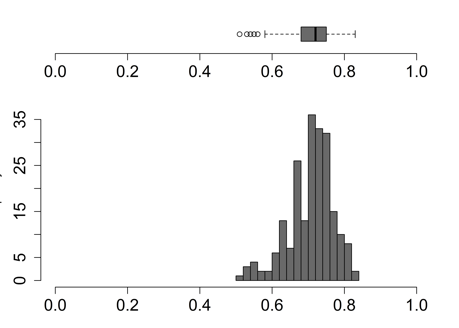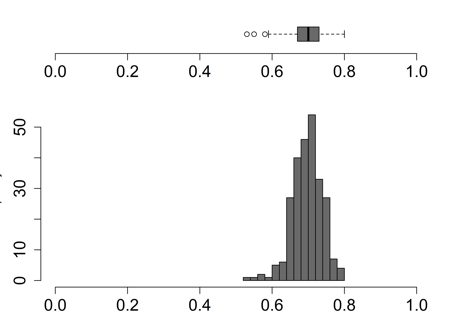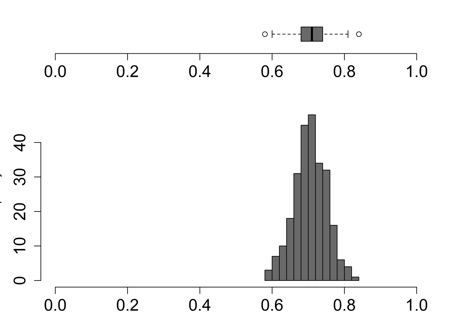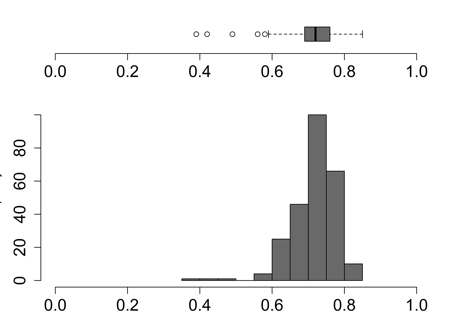 | | | |
| NDVI_4 | 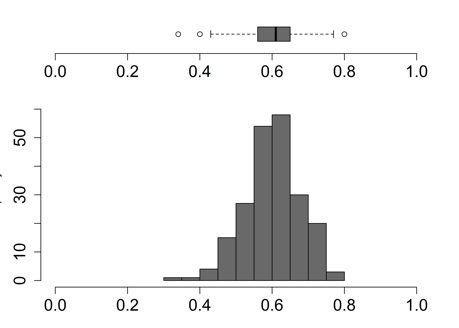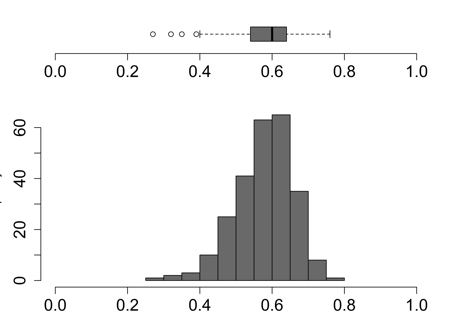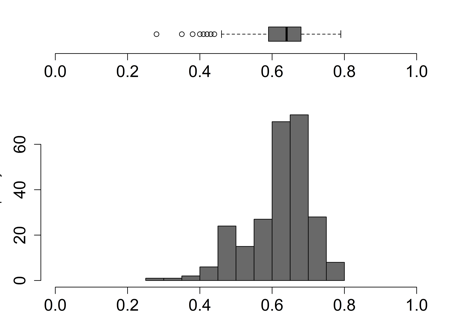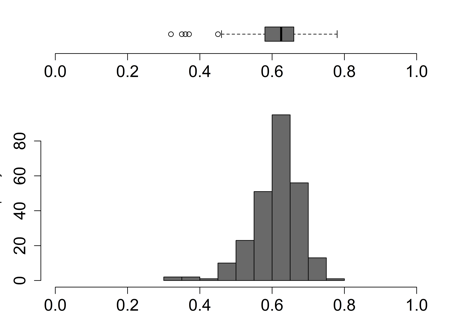 | | | |
| NDVI_5 | 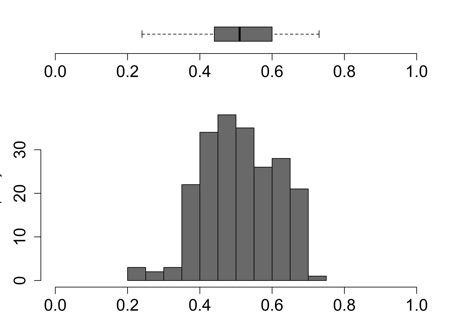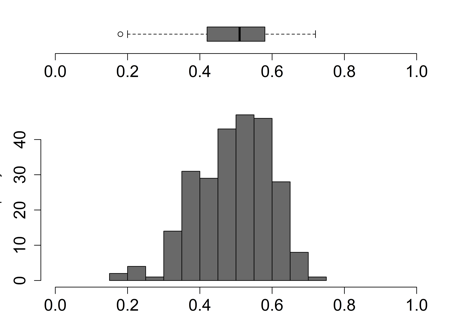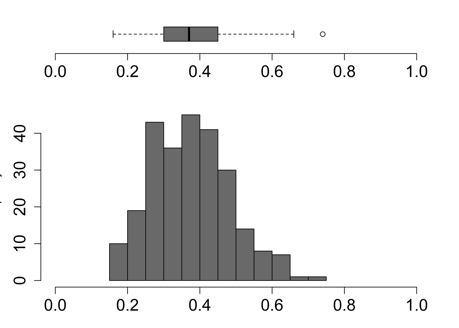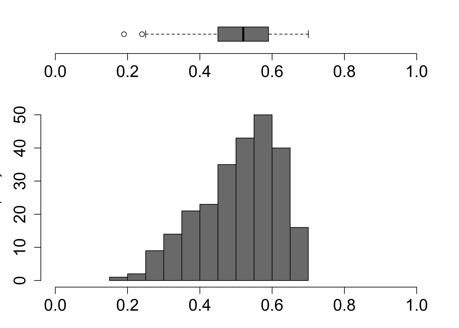 | | | |
| NDVI_6 | 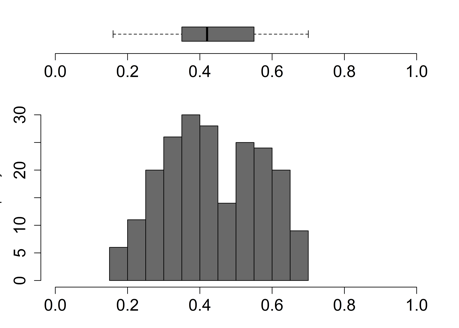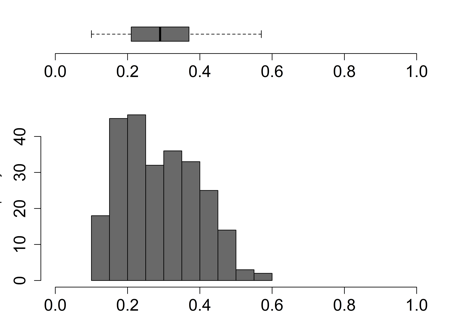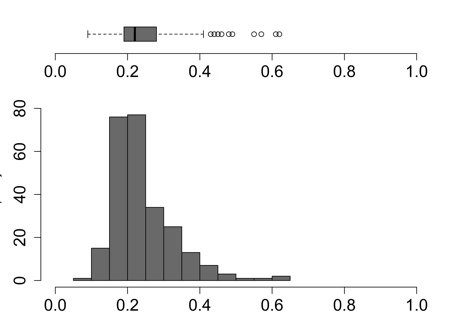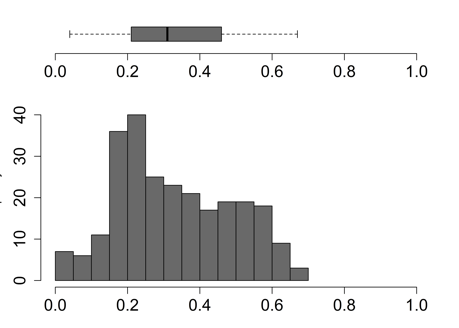 | | | |
| RS | 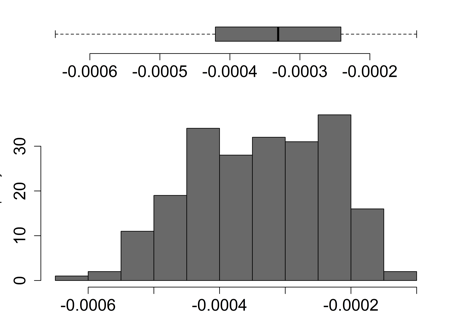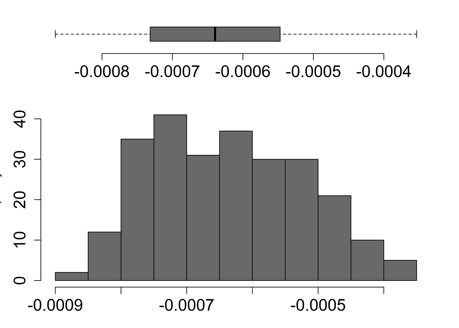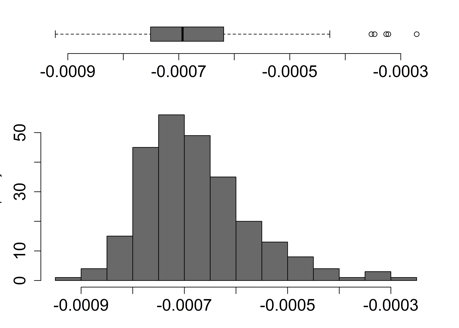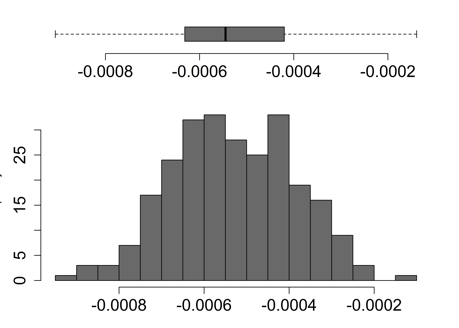 | | | |
| SG | 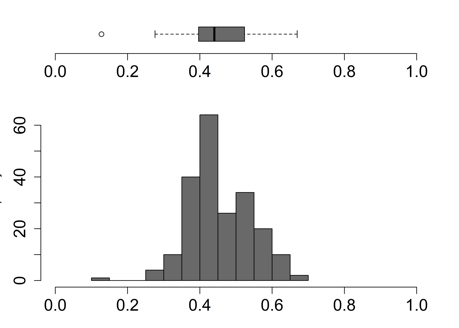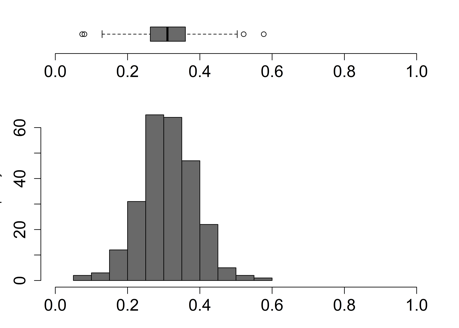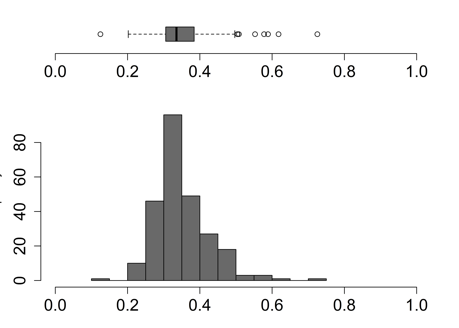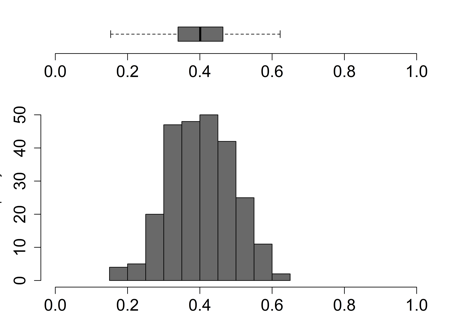 | | | |
